# The educational gradient of obesity increases among Swedish pregnant women: a register-based study

**DOI:** 10.1186/s12889-015-1624-6

**Published:** 2015-04-01

**Authors:** Helena Bjermo, Simon Lind, Finn Rasmussen

**Affiliations:** Unit of Child and Adolescent Health, Centre for Epidemiology and Community Medicine, Stockholm County Council, Box 1497, SE-171 29 Solna, Sweden; Child and Adolescent Public Health Epidemiology, Department of Public Health Sciences, Karolinska Institutet, SE-171 77 Stockholm, Sweden

**Keywords:** Obesity, Overweight, Pregnancy, Socioeconomic factors, Education

## Abstract

**Background:**

Overweight or obesity is detrimental during pregnancy. We studied time trends in the educational gradient of overweight and obesity among pregnant women. Differences in overweight and obesity by area of residence and country of birth were also examined.

**Methods:**

The study was based on the Swedish Medical Birth Register between 1992 and 2010 and included 1,569,173 singleton pregnancies. Weight and height were registered during the first visit at the antenatal-care clinic. Data on education, country of birth, and area of residence were derived from registers with national coverage.

**Results:**

In 2008–2010, 32% of Swedish nulliparous pregnant women were overweight or obese. The relative risk of obesity among lower educated women compared to women with higher education increased from 1.91 (95% confidence interval: 1.85-1.97) in 1992–1995 to 2.09 (95% confidence interval: 2.05-2.14) in 2008–2010. There was an inverse linear relationship between risks of overweight or obesity, and population density and type of residence municipality. An excessive gestational weight gain according to the American Institute of Medicine was observed among 57-63% of the overweight or obese women, but there were small differences by education. Pregnant women born in Africa, Middle East or Latin America had higher risks of being overweight or obese compared to women born in Sweden.

**Conclusions:**

The prevalence of obesity as well as the social inequalities in obesity during pregnancy increased in Sweden between 1992 and 2010. Further understanding of social inequalities and geographical differentials in health behaviours of pregnant women is needed when planning public health interventions.

## Background

As obesity has taken epidemic proportions, higher prevalence of obesity is observed also in pregnant women [1-3]. Maternal overweight or obesity during pregnancy can lead to several adverse outcomes for both mother and foetus. Obese women are more likely to have gestational diabetes mellitus, hypertension, pre-eclampsia, other obstetric complications and caesarean section [1,4,5]. Pre-pregnancy obesity is further associated with increased risk of adverse pregnancy outcomes such as stillbirth [6], preterm delivery [3], birth injuries [7], and that the new-borns become large for gestational age [1,4,5] with subsequent increased risk for childhood obesity [1]. Maternal gestational weight gain (GWG) is also related to these adverse pregnancy and birth outcomes [8,9], including an increased risk of macrosomia at birth [9], and increased risk of childhood obesity [10,11]. Thus, obesity in early pregnancy and also excessive GWG are known to have consequences for pregnancy and birth outcomes and the risk of childhood obesity in the next generation.

It is well known that individuals with lower education and socioeconomic position are more often overweight or obese in comparison to people with higher education or socioeconomic position. This social inequality is also observed in pregnant women [5,12,13]. A steepened social gradient has been implied in the Nordic countries but the evidence for increased social inequality of overweight and obesity is inconsistent [14]. Thus, further knowledge about time trends of socioeconomic inequalities in overweight or obesity is needed.

An urban–rural gradient of obesity has also been observed [12,15,16], with lower risk of obesity in urbanized areas. Among Swedish pregnant women, living in rural areas has been shown to almost double the odds of obesity after taking occupation and education into account [12]. Also in young Swedish males an urban–rural gradient is observed taking socioeconomic position, intelligence quotient and parental education into account [16]. The risk of obesity is further related to country of birth and data from the Swedish Medical Birth Register indicate that women with non-Nordic origin have higher prevalence of overweight [3], but more specific results on country of birth are lacking. Other European studies indicate higher pre-pregnancy BMI among women from African and Middle Eastern countries compared to the majority population in each country [17].

The main aim was to explore time trends of weight status among pregnant women in Sweden and to investigate if the educational gap in overweight or obesity has increased, decreased or remained stable during the last two decades. Additional aims were to explore differences in overweight and obesity according to area of residence and country of birth.

## Methods

### Study population

The study was based on the Swedish Medical Birth Register between 1992 and 2010. This is a national register including almost all pregnancies resulting in labour [18]. In 2010, 99.4% of all newborn were reported to the register [2]. Data are derived from medical records written at the antenatal health care centres, obstetric departments, and sometimes neonatal units. The present study includes singleton pregnancies with data on weight and height from the first visit to the antenatal-care (n = 1,569,173). Data on GWG were available for 38% (n = 596,699) of the pregnancies.

Weight and height were registered during the first visit at the antenatal-care clinic. This visit most often takes place between gestational week 8 to 12 [18]. According to the guidelines for antenatal care, weights registered in the medical records should be measured by the midwife. Thus, most weights are measured, even though some weights may be self-reported. GWG was calculated by subtracting weight at the first antenatal care visit from the weight at delivery. Body mass index (BMI) was calculated as weight (kg) divided by height (m) squared and categorised according to the World Health Organization (WHO) categories [19]: underweight (<18.5 kg/m^2^), normal weight (18.5-24.9 kg/m^2^), overweight (25.0-29.9 kg/m^2^) and obese (≥30.0 kg/m^2^). Maternal age was categorized into five groups: ≤24 years, 25–29 years, 30–34 years, 35–39 years, and 40 years or higher.

Data on education, country of birth, and area of residence were derived from the Longitudinal Integration Database for Health Insurance and Labour Market Studies (LISA) held by Statistics Sweden. Low education was defined as elementary school or high school, and high education as university level or doctoral degree. When used as a covariate, education was categorized into five levels: ≤ 9 years of elementary school, higher than 9 years of elementary school but less than 3 years of high school, 3 years of high school, higher than high school but less than 3 years of higher education, ≥ 3 years of higher education or doctoral degree. Area of residence was applied by an abbreviated version of a classification with seven categories mostly based on population-density but also on size and location of the municipalities (Table 1). The following categories were used in the abbreviated version: Stockholm area, Gothenburg area, Malmö area, larger municipalities, central district municipalities, densely populated municipalities, and sparsely populated municipalities. To investigate the magnitude of GWG, the recommendations of Institute of Medicine (IOM) [8,20] were used. The recommendations are as follows: 12.7-18.1 kg (for women who are underweight), 11.3-15.9 kg (for women who are normal weight), 6.8-11.3 kg (for overweight women), and 5.0-9.1 kg (for obese women). The study was approved by the Regional Ethical Review Board in Stockholm (“Regionala etikprövningsnämnden i Stockholm”), Sweden (Dnr 2011/591-31/5).

**Table 1:** **Definition of residential area categories**

**Residential area**	**Description**
Stockholm area	The capital city of Sweden and surrounding municipalities. Approximately 2 100 000 inhabitants live in the area.^b^
	Stockholm is located on the east coast of Sweden.
Gothenburg area	Sweden’s second largest city and surrounding municipalities. Approximately 960 000 inhabitants live in the area.^b^
	Gothenburg is located on the west coast of Sweden.
Malmö area	Sweden’s third largest city and surrounding municipalities. Approximately 620 000 inhabitants live in the area.^b^
	Malmö is located in the south of Sweden.
Larger municipalities	Municipalities with more than 90 000 inhabitants within 30 kilometres radius of the municipality centre. These municipalities are mainly located in the southern part of Sweden.^a^
	Municipality inhabitants range between 6 000 and 205 000.^b^
Central district municipalities	Municipalities with more than 27 000 inhabitants but less than 90 000 inhabitants within 30 kilometres radius of the municipality centre, and with more than 300 000 inhabitants within 100 kilometres radius of the municipality centre. These municipalities are mainly located in the southern part of Sweden.^a^
	Municipality inhabitants range between 4 000 and 64 000.^b^
Densely populated municipalities	Municipalities with more than 27 000 inhabitants but less than 90 000 inhabitants within 30 kilometres radius of the municipality centre, and with less than 300 000 inhabitants within 100 kilometres radius of the municipality centre.
	These municipalities are mainly located in the northern part of Sweden.^a^
	Municipality inhabitants range between 8 000 and 72 000.^b^
Sparsely populated municipalities	Municipalities with less than 27 000 inhabitants within 30 kilometres radius of the municipality centre.
	These municipalities are mainly located in the northern part of Sweden.^a^
	Municipality inhabitants range between 2 000 and 23 000.^b^

^a^Statistics Sweden (2003). Karta over H-regionernas omfattning [Area range of H regions].

http://www.scb.se/Grupp/Hitta_statistik/Regional%20statistik/Kartor/_Dokument/H-region_farg_karta.pdf [accessed 28 October 2014].

^b^November 1st 2013 according to Statistics Sweden (Statistics Sweden. Befolkningsstatistik [Vital statistics]. http://www.scb.se/sv_/Hitta-statistik/Statistik-efter-amne/Befolkning/Befolkningens-sammansattning/Befolkningsstatistik/25788/25795/Folkmangd-1-november---Kommun-och-riket/368232/ [last update: 11 December 2013, accessed 28 October 2014]).

Municipalities were divided according to local and regional population statistics. Population density of the table is sorted in a decreasing order.

### Statistical analyses

Linear regression models where used to calculate age-adjusted prevalence. Relative risks (RR) of overweight versus normal weight and obesity versus normal weight were estimated using Poisson regression with robust variance with the Genmod procedure in SAS 9.3. Childbearing is associated with weight gain, also in the long term [21,22], and pregnancy related weight retention is highly variable among women [23]. We stratified for parity and used only nulliparous women in the time trend analyses of overweight and obesity prevalence. These analyses were further adjusted for maternal age. To increase the power, all other relative risk estimates were based on all singleton pregnancies between 1992 and 2010. These relative risks were adjusted for maternal age, parity, education, year of birth, and country of birth when not used as the outcome. Relative risks of GWG above the IOM recommendations depending on education were calculated within strata of normal weight, overweight and obesity. Women gaining less than the recommendations were excluded.

## Results

### Time trends and the educational gradient

**Figure 1:**
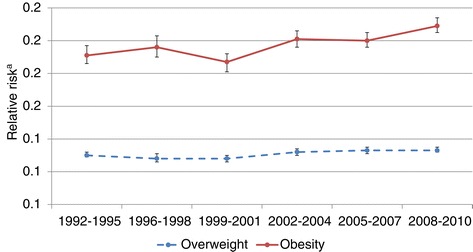
**Time trends in relative risks of overweight and obesity between Swedish nulliparous women with low vs high education.** BMI was assessed at the first visit to the antenatal-care clinic. ^a^Relative risk (95% confidence interval) for overweight/obesity among women with low education compared to higher educated women, adjusted for maternal age.

The overweight and obesity prevalence among all singleton pregnancies in 2008–2010 were 25% and 12%, respectively. Prevalence of overweight and obesity among nulliparous women at their first visit to the antenatal-care clinic between 1992 and 2010 are presented in Table 2. The prevalence of overweight and obesity has increased since 1992. During the 21^th^ century, the increase seems to have levelled off but an increase in obesity prevalence was still observed among lower educated women (Table 2). The relative risk of obesity among women with low education compared to higher educated women increased slightly from 1992–1995 to 2008–2010 (Figure 1). In absolute terms, the differences in prevalence between high and low educated women increased more than two-fold from 3.1% in 1992–1995 to 6.7% in 2008–2010 (Table 2).

**Table 2:** **Time trends in educational differences of overweight and obesity among nulliparous women in Sweden**

	**1992-1995**		**1996-1998**		**1999-2001**		**2002-2004**		**2005-2007**		**2008-2010**	
n(nulliparas)	138,713		87,809		91,825		107,112		114,122		129,717	
Overweight (25.0-29.9 kg/m^2^)												
All^a^	18.3	(18.0-18.6)	21.0	(20.6-21.3)	22.3	(22.0-22.7)	22.7	(22.4-23.0)	22.7	(22.4-23.0)	22.6	(22.3-22.8)
High education^a^	16.1	(15.7-16.5)	18.7	(18.3-19.2)	20.3	(19.9-20.7)	20.6	(20.2-21.0)	20.9	(20.6-21.3)	20.9	(20.6-21.3)
Low education^a^	20.6	(20.2-21.1)	23.6	(23.0-24.1)	24.8	(24.3-25.4)	25.6	(25.2-26.1)	25.4	(24.9-25.8)	25.6	(25.1-26.0)
Obesity (≥30.0 kg/m^2^)												
All^a^	5.1	(4.9-5.3)	6.7	(6.5-6.9)	7.8	(7.6-8.0)	8.8	(8.6-9.0)	9.3	(9.1-9.5)	9.6	(9.4-9.8)
High education^a^	3.6	(3.4-3.8)	4.7	(4.4-4.9)	5.9	(5.6-6.1)	6.4	(6.2-6.7)	7.0	(6.8-7.2)	7.1	(6.9-7.3)
Low education^a^	6.7	(6.4-7.0)	8.9	(8.5-9.3)	10.2	(9.8-10.5)	12.1	(11.7-12.4)	12.9	(12.6-13.3)	13.8	(13.4-14.1)

^a^Age-standardized prevalence (%) and 95% confidence interval.

BMI was assessed at the first visit to the antenatal-care clinic.

### Area of residence and country of birth

**Figure 2:**
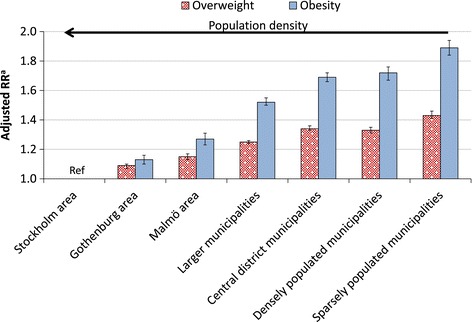
**Risk of overweight or obesity among pregnant women in relation to population density of residence municipality.** BMI was assessed at the first visit to the antenatal-care clinic. *RR* Relative Risk n: Stockholm area: overweight = 69 273, obese = 24 068, Gothenburg area: overweight = 32 892, obese = 11 814, Malmö area: overweight = 22 272, obese = 8 307, larger municipalities: overweight = 136 935, obese = 59 021, central district municipalities: overweight = 64 299, obese = 29 517, densely populated municipalities: overweight = 19 490, obese = 9 085, sparsely populated municipalities: overweight = 20 642, obese = 10 146. ^a^Calculated among all pregnancies between 1992 and 2010, and adjusted for maternal age, education, parity, country of birth, and year of birth.

There were large differences in overweight and obesity by type of residence municipality. The relative risks of overweight and obesity were inversely related to population density and women living in sparsely populated municipalities had the highest risk (Figure 2). Pregnant women born in Africa, Middle East or Latin America had higher risks of being overweight or obese compared to women born in Sweden with adjustment for level of education. In contrast, women born in Europe (excluding the Nordic countries), North America, Australia, New Zealand or former USSR (Union of Soviet Socialist Republics) had lower risk of overweight or obesity, also adjusted for education. Asian born mothers had the lowest risk of both overweight and obesity (Table 3). Similar results were obtained after adjusting for gestational week at the first antenatal care visit.

**Table 3:** **Risk of overweight or obesity among pregnant women in relation to country of birth**

**Country of birth**	**Overweight (25.0-29.9 kg/m** ^**2**^ **)**				**Obesity (≥30.0 kg/m** ^**2**^ **)**			
	**n**	**%**	**RR** ^**a**^	**(95% CI)**	**n**	**%**	**RR** ^**a**^	**(95% CI)**
Sweden	297,191	81.4	Reference category		124,563	82.1	Reference category	
Nordic countries (excluding Sweden)	8,407	2.3	1.04	(1.01-1.06)	3,648	2.4	1.10	(1.06-1.14)
EU15, North America, Australia, and New Zealand	3,423	0.9	0.89	(0.86-0.92)	1,396	0.9	0.91	(0.86-0.97)
Europe (excluding EU15) and former USSR	13,045	3.6	0.95	(0.94-0.97)	4,465	2.9	0.73	(0.71-0.75)
Africa (excluding North Africa)	7,357	2.0	1.21	(1.18-1.23)	3,911	2.6	1.17	(1.13-1.22)
North Africa and Middle East	17,676	4.8	1.30	(1.28-1.32)	7,450	4.9	1.14	(1.11-1.17)
Asia^b^	13,362	3.7	0.87	(0.86-0.89)	3,952	2.6	0.56	(0.54-0.58)
Latin America	4,686	1.3	1.16	(1.13-1.20)	2,321	1.5	1.27	(1.21-1.33)

*RR* Relative Risk.

*CI* Confidence Interval.

^a^Calculated among all pregnancies between 1992 and 2010, and adjusted for maternal age, education, parity, and year of birth.

^b^Melanesia, Micronesia and Polynesia were included in the Asian region: n(overweight) = 20, n(obese) = 12.

BMI was assessed at the first visit to the antenatal-care clinic.

### BMI in early pregnancy and total gestational weight gain

BMI in early pregnancy and total GWG by level of education among nulliparous women are shown in Table 4. Among all parities, 63% of the overweight and 57% of the obese women gained weight above the IOM recommendations. There were only minor educational differences in total GWG. However, among those with normal weight, women with a low education had a higher risk of excessive GWG compared to women with a high education, after adjusting for BMI at the first antenatal care visit, maternal age, parity, year of birth, and country of birth (adjusted RR: 1.08 [95% confidence interval (CI): 1.07-1.09]). In contrast, lower educated overweight or obese women had slightly lower risk of excessive weight gain than women with higher education (adjusted RR: 0.98 [95% CI: 0.97-0.99], and adjusted RR: 0.98 [95% CI: 0.96-0.99], respectively).

**Table 4:** **BMI in early pregnancy and total gestational weight by level of education in Swedish nulliparous women**

		**BMI in early pregnancy (kg/m** ^**2**^ **)**	**Total gestational weight gain (kg)**
	**n**	**Mean (SD)**	**Mean (SD)**
Normal weight (18.5-24.9 kg/m^2^)			
Low education	196,252	22.1 (1.7)	14.0 (5.1)
High education	182,277	22.1 (1.6)	13.7 (4.5)
All	378,529	22.1 (1.7)	13.8 (4.8)
Overweight (25.0-29.9 kg/m^2^)			
Low education	83,103	27.0 (1.4)	13.4 (6.0)
High education	56,222	26.9 (1.4)	13.6 (5.5)
All	139,325	27.0 (1.4)	13.5 (5.8)
Obesity (≥30.0 kg/m^2^)			
Low education	41,068	34.0 (3.8)	10.7 (6.8)
High education	18,461	33.5 (3.5)	11.2 (6.5)
All	59,529	33.9 (3.7)	10.9 (6.7)

*SD* standard deviation.

## Discussion

The prevalence of overweight and obesity among Swedish pregnant women has increased since 1992, especially among those with low education. Women with low education or living in sparsely populated areas are at higher risk of obesity in early pregnancy.

In 2008–2010, 25% of Swedish pregnant women were overweight and 12% were obese at their first visit to the antenatal care clinic. This is slightly higher than pre-pregnancy weights observed in other countries [24-27]. Discrepancies are that most previous studies investigated somewhat older data, and that weight was assessed early in the first trimester instead of pre-pregnancy weight in the present study. A GWG rate of 0.22 kg per week has previously been estimated during the first trimester [28] and the most frequent time point for the initial visit to the antenatal clinic is at ten weeks of gestation. If a 2.2 kg weight gain before the first antenatal care visit was anticipated for all participants, estimated pre-pregnancy obesity prevalence among nulliparous women was 1.0-1.6 percent units lower than the prevalence observed in Table 2. According to the Danish Medical Birth Register, 21% of the pregnant women had overweight and 12% had obesity during 2004–2010 [24]. In the Norwegian Mother and Child Cohort Study conducted between 1999 and 2008, 22% of the women becoming pregnant were overweight and 9% were obese [25].

Our results showed a slower increase in prevalence of obesity during the 21^th^ century, which is in accordance with previous findings in Swedish populations [29-31]. However, Swedish data on whether the socioeconomic gradient of obesity is changing are inconsistent [14]. A previous Swedish study has indicated a narrowing of the socioeconomic gap for adults aged 25–44 years between 1988–1989 and 1996–1997, whereas it seemed stable for adults 45–64 years [32]. In contrast, a higher increase in prevalence of obesity in lower social classes has been suggested in Stockholm County during the 21^th^ century [30]. Increased socioeconomic gap in overweight and obesity has also been shown among young Swedish men between 1970 to 2000 [33]. An increased socioeconomic difference in obesity prevalence from 1990–1995 to 2002–2007 was further observed in the North of Sweden [29]. Using education as a measure of socioeconomic position, the present study indicates that the socioeconomic gap in obesity is increasing, both in relative and absolute terms. In contrast, the socioeconomic difference in overweight seems to be stable over time. We can only speculate in why the socioeconomic gap of obesity appears to increase but the increasing physical activity in Sweden seems to be mainly among individuals with higher education [29]. Further, individuals with higher education may be moving to urbanized areas, with its beneficial contextual factors, to a higher degree [29]. It may also be speculated that individuals without university education are more excluded from the labour market today or that health literacy has become more central in the health care system.

In line with other Swedish studies [12,15,16,29], an urban–rural gradient of overweight and obesity was observed among pregnant women independently of educational level. The sample size in the present study allowed for a more detailed categorisation of residential area than the often used urban/rural classification. Interestingly, the risk for overweight or obesity seems to increase linearly with decreasing population density of municipality of residence based on comparisons of risk ratios. Possible explanations may be that habitants in rural areas are more dependent on motorized transportation and higher accessibility to training facilities in urbanized areas. Also other contextual factors could contribute, such as that a larger part of the population is highly educated in urbanized areas, irrespective of the own educational level.

Our results show both increased and decreased risks of overweight or obesity in different groups of non-Swedish born pregnant women compared to Swedish born pregnant women. Women born in Africa, the Middle East, and Latin America had higher risk compared to native Swedes, whereas women born in other countries had lower risk. These risks remained after adjustment for education. Other studies [27,34,35] confirm ethnical differences in obesity prevalence, especially among immigrants from Middle East or Latin America. Compared to native Swedes, more than twice as high obesity prevalence has been observed among women from Middle East [35], Turkey [34] and Chile [34]. Likewise, in a Dutch study [27], ethnical differences in GWG and postpartum weight retention were observed, with Turkish women being at higher risk for retaining weight. Women born in other Nordic countries than Sweden had a slight over risk of obesity. Previous studies have shown higher BMI and body fat percentage among Finnish-born women compared to native Swedes [36,37]. Possible factors explaining the differences in obesity between countries of birth could be socioeconomic inequalities, difficulties to be included on the labour market, diverse lifestyle, genetic susceptibility, language barriers and cultural attitudes to sickness and health care [35].

Large proportions (57-63%) of the overweight and obese women gained more in weight during pregnancy than recommended by the IOM [8,20] and were at increased risk of short-term and long-term complications to the mother and the offspring during pregnancy, delivery and the post-term period. Our results indicate that further research is needed on evaluations of primary prevention interventions which might be implemented in routine antenatal care to limit excessive GWG among Swedish pregnant women. While the educational differentials in body weight in early pregnancy were strongly inversely related to maternal level of education, GWG showed less strong associations. In line with ours, a recent study showed that excessive GWG was more prevalent among low educated normal weighted women whereas education did not seem to protect against excessive GWG among overweight and obese women [13]. In fact, we observed a slightly higher risk of excessive gestational weight gain among higher educated overweight or obese women, also after adjusting for pre-pregnancy BMI.

The present study has strengths and limitations. It is a strength that it is nationwide by including almost all pregnancies progressing to labour in Sweden during the investigated time period. Due to the large number of women, we had sufficient power to investigate residential area and country of birth at more detailed levels. Since nearly 75% of the Swedish women have at least one child [22], the results are believed to be generalizable to the fertile Swedish female population. The study is limited by that the Swedish Medical Birth Register does not contain data on weight before conception. Weight was assessed at the first visit to the antenatal care clinic. Most of these visits are scheduled between gestational week 8 to 12 [18].

## Conclusions

The prevalence of obesity as well as the social inequalities in obesity during pregnancy increased in Sweden between 1992 and 2010. Pregnancy is thought to be a susceptible period where motivation for risk-reducing behaviour changes is higher [38]. Antenatal care could therefore be a suitable arena for preventive interventions. Such interventions could benefit both mothers and their children in the short as well as long term. To reduce social inequalities in maternal gestational overweight and obesity, emphasis should be on understanding health behaviour change and health literacy in different socioeconomic and ethnic groups and how effective primary and clinical interventions might be implemented in primary and antenatal care settings.

## Abbreviations

BMI: Body mass index
CI: Confidence interval
GWG: Gestational weight gain
IOM: Institute of Medicine
RR: Relative risk
WHO: World Health Organization
