# Erratum to: ‘The educational gradient of obesity increases among Swedish pregnant women: a register-based study’

**DOI:** 10.1186/s12889-016-3156-0

**Published:** 2016-06-15

**Authors:** Helena Bjermo, Simon Lind, Finn Rasmussen

**Affiliations:** Unit of Child and Adolescent Health, Centre for Epidemiology and Community Medicine, Stockholm County Council, Box 1497, SE-171 29 Solna, Sweden; Child and Adolescent Public Health Epidemiology, Department of Public Health Sciences, Karolinska Institutet, SE-171 77 Stockholm, Sweden

Unfortunately, the original version of this article [1] contained an error. The presentation of Fig. 1 was incorrect. The numbers on the Y-axis are incorrect and need modification. The correct version is provided below.

**Fig. 1:**
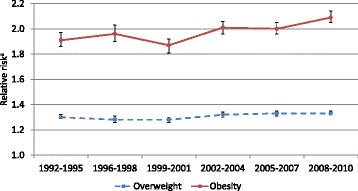
Time trends in relative risks of overweight and obesity between Swedish nulliparous women with low vs high education. BMI was assessed at the first visit to the antenatal-care clinic. ^a^Relative risk (95 % confidence interval) for overweight/obesity among women with low education compared to higher educated women, adjusted for maternal age
